# The Treatment of Appendicitis.—A Conservative View.—Illustrative Cases1Read before the Alumni Association of Niagara University, May 2, 1892.

**Published:** 1892-08

**Authors:** Nelson G. Richmond

**Affiliations:** Fredonia, N. Y.


### Buffalo Medical and Surgical Journal

Vol. XXXII. AUGUST, 1892. No. 1.

### Original Communications.

### THE TREATMENT OF APPENDICITIS.—A CONSERVATIVE VIEW.—ILLUSTRATIVE CASES.[1]

By NELSON G. RICHMOND, M. D., Fredonia, N. Y.

There is little doubt that in choosing the above title, or more particularly in reaching the conclusions which the title suggests, much adverse criticism will be aroused. I am perfectly well-aware that there has been no subject more fully or profitably discussed during the last three years than this one—appendicitis. The popular side of the question today is surgical procedure. The record of the operators is a brilliant one. Very many lives have unquestionably been saved, which would have been sacrificed under more conservative methods. Surgery is necessary for very many cases.

The reason for urging a conservative view in the light of the above facts is this: The operators have been so carried away by their own results, that they are led to advise against *all* medicinal measures.

By withholding mechanical treatment and medicinal measures, which should have been resorted to at the proper time, there is little doubt that many cases will require the surgeon to relieve them later on.

What then does A Conservative View of the Treatment of Appendicitis mean? It means active interference with the progress of the cause of the disease. It means removal of the cause. It means going to work *immediately*, with the hope of getting the patient beyond the point of danger by the time the surgeon, who has been watching *his* case, and advising against any interference, is getting ready to operate.

In the treatment of disease, the first thing to be taken into consideration is the cause. The causes, of appendicitis have a direct

1. Read before the Alumni Association of Niagara University, May 2, 1892.

bearing upon the treatment, and, familiar as they are, should be considered in this connection.

They are: (1) The lodgment and impaction of fecal matter in the appendix and cecum, one or both. This is the most common. (2) Hard, indigestible, or foreign bodies, which produce inflammation and sometimes perforation by their pressure. (3) The overloading of the stomach.

Fitz found that fecal concretions comprised forty-seven per cent. of the entire number of cases he had collected; foreign bodies only twelve per cent. of the entire number.

There is a difference of opinion as to the seat of the primary lesion. Some claim it to be the appendix, others the cecum. One says that, in comparison, the cecum plays no part at all. This point is an important one to consider if we take into account two varieties of appendicitis: One, the stercoreal variety; the other, where more acute, inflammatory symptoms seem to arise in the appendix, resulting in early perforation, or possibly gangrene.

How can one consider carefully the above causes, viz., impaction of feces; hard, indigestible, or foreign bodies; the overloading of the stomach,—without at once setting to work to remove them? We doubt not the active, busy practitioner does attempt to remove them first of all. But here is what some of the men we consider as authority say on the subject:

McBurney has a most brilliant record as an operator. His name is in the front rank, associated in the investigation of this disease with Fitz, Senn, Treves, Stimson, Weir, Price, and Keen. The point of tenderness, from one to two inches inside the right anterior superior spinous process of the ilium, on a line drawn from the spine to umbilicus, is now universally known as "McBurney's point." He claims it to be almost a pathognomic sign in the diagnosis of this disease.

McBurney says: "Laxatives should be carefully avoided, and enemas as well, that all peristaltic action may be discouraged."

J. Lewis Smith advises postponing laxatives, or laxative enemata, until tenderness and inflammatory symptoms have subsided by the use of opium and an ice-bag. Afterwards, if indicated, he advises an enema,—calomel or castor oil.

Volz strongly insists upon opium treatment, and warns against the use of cathartics or enemata.

Matterstock cannot caution too earnestly against any attempt to relieve constipation by either cathartics or clysmata.

Keen says nothing about the medical treatment in a paper consulted, but does say this: "I earnestly believe that operation is rightly undertaken when there is persistent pain and tenderness, especially at McBurney's point, with even slightly increased resistance without any tumor, with possibly a slight edema and a moderate fever." He does express himself, however, as condemning operative interference in the mild form of appendicitis. Most of such attacks, he thinks, have been overlooked, or have been called simple indigestion, colic, or some intestinal disorder.

Dr. Pepper advises against the use of laxatives.

Finally, an editorial in the *Medical Record*, commenting on a paper of Dr. Musser, of Philadelphia, says: "Treatment consists of abstinence from food, and absolute avoidance of interference with the state of the bowels."

The above authorities are quoted to show what these men advise concerning laxatives and enemata, and that there is some excuse for the writing of this paper.

Let us now consider the advice of some surgeons more conservative in their views. The name of Treves in England is associated with appendicitis as indelibly as that of Fitz in the United States. His name, moreover, is suggestive of boldness. At a gathering of physicians, the so-called "medical treatment" had produced a smile on the faces of those present. Yet, he says, medical treatment is successful in very many cases; and to treat all these cases surgically would only lead to disaster. The great majority admitted into the London Hospital during the last ten years recovered under it. No doubt, the epidemic of removal of appendicitis, which had prevailed in some quarters, had been extreme.

He seldom found it necessary to make an incision before the fifth day, and did not consider it advisable to do so then, unless there were urgent symptoms. In most cases it would only be considered after the first week.

This is quite different from the counsel of McBurney, who advises laparatomy within thirty-six hours in case there is no amelioration of symptoms.

In the April 16, 1892, number of the *Medical Record* is a paper of a remarkable experience by Dr. McBurney. He presents the histories of five cases of appendicitis operated upon between December 6 and December 21, 1891, all of them being successful. No one can read the histories of these cases and criticise the wisdom or necessity of operating. They all belonged to the recurrent, perforative and gangrenous variety. In this same paper he answers the criticisms of Treves and Pepper, some of which I have quoted above.

Treves found, as a result of his investigations in 1889, that there was no cellular investment around the cecum. He also determined that the peritoneum was absent on the post-surface of it. Hence, the terms *peri*typhlitis and *para*typhlitis were misnomers. They have since been discarded. Fortunately, typhlitis, too, has given way to the more suggestive and sensible name *appendicitis*.

Weir only advises operation in serious cases, or cases of frequent recurrence. He admits that the milder lesions of an inflamed or stenosed appendix which the physician meets, the surgeon may be unacquainted with. "For, after all," he says, "laparatomy, however carefully conducted, is an operation of risk."

It was my good fortune to be present at the meeting of the State Medical Society at Albany, February, 1891, when a whole evening's discussion was given to the subject, Appendicitis. The Pathology was ably handled by our own Dr. Mynter, together with Gerster, of New York. The Indications for Early Laparatomy in Appendicitis was the subject of papers by McBurney and Keen. The Technique of Operative Interference in Appendicitis was considered by Stimson and Fowler. The Propriety of and the Indications for the Resection of the Appendix Vermiformis During the Quiescent Stage of Chronic Relapsing Appendicitis was the title of papers by Price and Weir. Dr. Bacon, of New Haven, had confined his operative experience to the abscess stage.

The conclusions reached by those who listened to this discussion might be as varied as the character of the listeners, but the conclusion I arrived at was that the position taken by Dr. Weir or Dr. Bacon was the one to follow in everyday practice.

Dr. Bacon had operated on over forty cases in the abscess stage, losing only two. Imagine, if you can, the number saved the operation, from which these forty were taken, and you get an idea of the actual percentage of recoveries from this conservative method.

Dr. Weir, as stated above, would advise operation in cases of serious and frequent recurrence, only when the recurrence threatened life or incapacitated the patient for his chosen life-work.

Physicians in general practice, particularly those outside the large cities, find that patients do not submit willingly to an operation, of whatever character, without the very best of reasons. It is apt to be a question of life or death, not a matter of convenience or possible future necessity, which convinces them. Invariably they will take the chances on the future possibility. They have more faith in the future, and stand less in awe of the physician or surgeon. The surgeon of the larger cities does frequently what would be perfect suicide to the reputation and practice of the physician of the village. Hence, the advice which is of service to the physician or surgeon is the advice which he can follow. Some advise the operation in all cases of catarrhal or ulcerative appendicitis,—happily not all.

Dr. William T. Bull, in 1890, reported having seen five or six cases of serious ventral hernia from the operation.

Dr. A. H. Smith, the same year, reported six cases which resulted in suppuration. Three without operation recovered; three died notwithstanding an operation. He believes that every attack results in extra-fibrinous exudation, and less danger of abscess, should it form, rupturing into the peritoneum. This opinion does not necessarily clash with that of Dr. Weir.

In reviewing some of the literature on this subject, it has been my aim not to give opinions or quote authorities further back than the past three years. The discarding of the terms formerly used to designate it, the modern pathology, the more discriminating diagnosis, and the remarkable success of the operators, make the consideration of appendicitis a very different subject from that of "perityphlitis" of five years ago.

We now come to the treatment proper of appendicitis as met with in general practice. It is very simple. It has been foreshadowed throughout the discussion of this paper. It is the same whether the exciting cause seems to be from an overloaded stomach or from constipation. A history of constipation is not, however, necessary for the presence of a fecal accumulation. The bowels may have moved regularly every day, yet the amount of fecal matter obtained by judicious treatment is invariably surprising. This is easily accounted for; a portion of the stool being retained each day, notwithstanding the larger amount evacuated. It will take only a few weeks for the colon to become well distended, while a hard, dry impaction of fecal matter is lodged at the ileo-cecal valve.

First of all things necessary, then, is abstinence from food. The observers say low diet. I should say no diet. No more fecal matter should pass down to the ileo-cecal valve and lodge while it is obstructed.

Second, enemata should be administered. In urgent cases I usually say every two hours. Frequently these are kept up for four to five days. These enemata are useful for two reasons. First, to relieve pain. Second, to remove the obstruction. I know of no more efficient measure for the relief of abdominal pain, especially when it is low down, than hot enemata. It is often quicker in its action than a hypodermic of morphia, and will sometimes succeed when the latter fails. The enemata may be varied in character as fancy or experience may dictate, from castile soap-suds and warm water, with turpentine or castor oil added, to the infusion of lobelia, or even sale molasses.

Third, the judicious use of morphia, hypodermically or by mouth, as seems advisable, as a necessity, not as a curative measure. It should never be used to overcome peristalsis, as formerly advised, but simply for the relief of pain. It is too much to ask these patients to suffer, as they must for days and nights, without giving them some temporary relief. Each dose that is given, however, should be with the full knowledge of the fact that by locking up the secretions and delaying the action of the bowel, by so much time is the cure of the disease postponed. Nevertheless, it is equally true that by relieving pain the strength of the patient is preserved.

Fourth, castor oil is to be given when, after a few hours or a day, the pain is unrelieved, or the enlargement in the right iliac fossa is undiminished. Inflammatory action, I do not believe, contraindicates it, since the obstruction of the bowel by fecal matter is a common cause of the inflammation. It is my usual custom to repeat it in tablespoonful doses every six hours, until all fecal matter is removed from the bowel. This stage is usually marked by a number of offensive, diarrheic stools ; finally, green in color, and independent of an enema. In case a foreign body or undigested material is the cause of the trouble, this will usually precede the diarrhea. Castor oil is the only laxative which it seems wise to give. If it does not move the bowel, it at least does no harm. All other laxatives may. In one case fourteen tablespoonfuls of castor oil were given within four days, enemata being administered every two hours, day and night, for the same period, before the obstruction was removed.

The fifth and last measure of treatment is the application of hot flaxseed poultices, begun with the beginning of pain, and continued as long as inflammatory exudation exists.

The only authorities consulted who seem to approach the median ground of medicinal measures, are the following:

Henoch advises the opium treatment; castor oil or calomel for prolonged constipation or fecal accumulation; the ice-bag over cecum; and *when pain and tenderness cease*, a simple enema, or dose of castor oil.

Fenger, in the Cyclopedia of the Diseases of Children, says:

> If a certain diagnosis can be made between stercoreal typhlitis and perityphlitis (he retains the old name), the question is readily solved, and cathartics and clysmata will be as beneficial in the former as opium or morphia in the latter case.

As a student in medicine I remember reading Dr. William T. Bull's inaugural thesis, written in 1872, on Perityphlitic Abscess. He had collected sixty-seven cases. It is needless to say I had a very vague idea of the disease. All the cases, however, were treated during the first eight years of my practice, as outlined in this paper, and were successful. There were no histories kept, and no lasting impression was made. In September, 1888, I recorded my first and only fatal case, and the only case which ever came under my own observation where the inflammation and obstruction at the cecum being relieved, recovery did not follow. The exciting cause, too, was overeating. Evidences of abscess and symptoms of collapse came on, the patient dying the eighth day. Two other fatal cases, about this time, came within my knowledge. I do not know what treatment was pursued.

Autopsy revealed a large accumulation of pus; an appendix perforated in several places, and within, a concretion, size of a cherry stone, hard and resisting as the latter, which it was thought to be. The family secured it, however, and it could never be obtained for thorough examination. It might have been a gall-stone, but more probably was a fecal concretion. There is no doubt but that an operation was called for in this case. But owing to the condition of the patient and his surroundings, poverty-stricken in the extreme, it is very doubtful if it would have been successful.

My cases are fifteen in number, six females and nine males, varying in ages from nine to eighty years. They were of all the stages of intensity, from the cases with unmistakable symptoms, but relieved in two or three days, to the ones of several weeks' duration, marked by relapses, general peritonitis, and final evacuation of pus per rectum.

Several were watched very closely with the view that laparatomy would probably be called for. But with the natural conservatism prevalent outside the large cities, the time did not arrive when it seemed wise to interfere by operative measures.

They all recovered. All of the lighter, as well as the moderately severe cases, dated the amelioration of symptoms and beginning of recovery from the time stools were obtained which came from the ileo-cecal obstruction. These went on uninterruptedly towards recovery.

In two of the severest cases this period of sudsidence was followed by symptoms showing local or general peritonitis. Whether this was due to an extension of the original inflammation or to a fresh attack of peritonitis, caused by exposure and chill, it was hard to decide. In the severest case it seemed due to the latter.

They were all treated as outlined previously. Accumulation at the ileo-cecal valve with symptoms of local peritonitis was treated very actively, the tumefaction and peritonitis subsiding coincidently with the relief of the obstruction. When the peritonitis was general, stools having been already obtained, active treatment was omitted until the inflammation subsided.

As stated in the beginning of this paper, it is not my desire to decry operative measures. Laparatomy is necessary in very many cases. The chief aim has been to show that too many cases recover under medicinal measures to make it advisable to withhold such measures, as many of the operators quoted advise.

CASE I. Mrs. E. B., *æt.* 23; December, 1889. Found her suffering intensely from pain in the right iliac region. There was an elongated tumor in the ascending colon, very sensitive to the touch, and accompanied with considerable local peritonitis, fever, etc. Under the care of another physician, opiates had been given, laxatives and enemata had been enjoined, and she was growing rapidly worse.

Ordered hot enemata every two hours; ol. ricini in half-ounce doses every six hours, and morphia as required from necessity. This treatment was kept up for three days, small actions being obtained. Finally, a number of offensive stools, green and grumous in character, were accompanied by some hard, indigestible substances, which proved to be the gristle of meat. Rapid recovery. No trouble since.

CASE II. J. C., *æt.* 37; January, 1890. Pain, requiring opiates; fever, tenderness, and enlargement in right groin the size of two fists. Referred to previously as having taken fourteen tablespoonfuls of castor oil, and enemata every two hours for four days, before the colon was cleared out. Improvement dated from the relief of this symptom. No trouble since.

CASE III. George T., *æt.* 37. Ill in January, 1890. Enlargement in right iliac region, from obstruction and inflammatory exudation; great pain and tenderness, requiring frequent hypodermics of morphia; fever, emesis. Treatment: Hot enemata every two hours; castor oil every six hours, and hot applications. After the evacuation of the lower bowel, the stools began to have a fecal odor the third day. Marked relief was obtained the fourth day, following several diarrheic stools of foul odor and more or less green color. Perfect recovery.

CASE IV. was that of a little girl, nine years old. She was ill five days in January, 1891. In February there was a recurrence, she never having been entirely well. The second attack was marked by large exudation and considerable peritonitis. The disease left her weak and emaciated, and at one time suppuration was suspected, but it did not prove to be the case. Inflammatory symptoms subsided, and improvement in each attack began coincident with the relief of the fecal obstruction.

CASE V. was that of an athletic young man of 20 years. He had had five previous attacks, three being about the same in severity as the present one, and two lighter in character, He had the usual symptoms of fever, severe pains, local peritonitis, and an oblong tumor in the right iliac region. He was relieved the fourth day, after free evacuation of the bowel by the usual method.

CASE VI. Susie S., *æt.* 18. First attack March, 1891. Only two days in duration. Second attack in October, 1891. It took repeated doses of morphia to make the pain bearable. Local symptoms very marked; tenderness at McBurney's point; gradually developing tumor and general peritonitis. Relief the fourth day, from the usual treatment, and satisfactory convalescence.

CASE VII. M. T., male; *æt.* 38. Occurred in June, 1891. This case was unusually severe in its onset, and throughout its entire course. Had had appendicitis a few years before. He begged me to give him a large hypodermic of morphia, saying that it seemed to avert a previous threatened attack. Remaining with him a greater part of the night, and finding that morphia per orem, in repeated doses, failed to relieve the intense pain, he was given a hypodermic in addition.

It was necessary to keep him under morphia during the whole period. Tenderness, first at McBurney's point, was finally distributed over the entire area of thickening and enlargement, which extended from the anterior superior spinous process of the ilium to umbilicus and down to the middle of Poupart's ligament. There was fever, flushed face, sunken eyes, and persistent vomiting for days, first of the contents of the stomach, then of a green matter, and finally of that which was of the color of coffee grounds and had a fecal odor.

For several days I stood ready to operate. expecting to find at each visit the remaining symptoms which would justify the operation. Finally, when the stools showed that all accumulation at the ileo-cecal valve had been removed, vomiting ceased, he began to improve, and made a satisfactory convalescence.

Once since, he had symptoms precisely similar to those at the beginning of above attack. Giving a hypodermic of morphia, which put him to sleep and kept him so the remainder of the night and well into the next day, the attack was averted. It seemed to be one of perforative appendicitis.

CASE VIII. Mrs. G., 60 years of age. Complained for days of constant pain on the right side. Examination revealed tenderness at McBurney's point. Laxatives and enemata brought away hard, scyballous masses. The pain and tenderness was relieved.

CASE IX. Wm. A., *æt.* 35. Coming home one evening hungry, he ate a full box of sardines and drank a bottle of beer. Was taken with severe pain in iliac region before morning. He had fever, tenderness at McBurney's point, with symptoms of obstruction and inflammatory exudation surrounding this region.

The usual treatment relieved him, and he has had no recurrence since.

CASE X. Gay T., *æt.* 21, was taken in January, 1892, with pain in the right iliac region, which only a hypodermic of morphia would quiet. It was necessary to repeat this at regular intervals during the whole sickness. There was tenderness at McBurney's point, and an accumulation at the ileo-cecal valve. Inflammation came on, and exudation resulted in hardness and resistance to touch, and tenderness over entire region, even beyond umbilicus. Vomiting was persistent for three days. Knowing that he was accustomed to put no restraint on his unusually hearty appetite, enemata were begun immediately, each administration of them giving temporary relief. Castor oil was given in ounce doses. The fourth day relief came coincident with numberless stools, offensive in odor, tarry in consistence, and followed by green diarrhea stools for several days.

In a few days all inflammatory exudation had subsided, although tenderness was perceptible for some time.

CASE XI. was that of a married man of 23. He was taken ill in Buffalo, while on a business trip in February, and attended by a physician who staid with him several hours, and evidently understood the seriousness of the case.

On reaching home, the pain was still severe, and the first visit revealed the symptoms of appendicitis. He was a merchant by occupation, and was accustomed to eat his supper at the back of the store and wait on customers at the same time. The routine treatment was begun, the pain requiring hypodermics of morphia throughout the acute stage. He began to get relief the fourth day, and was improving when a relapse occurred, which appeared to be due to getting cold. Inflammation set in with increasing severity, the tongue became dry and red, hardness extended over entire space beyond umbilicus and median line, there being also general peritonitis as well. The eyes were sunken, features pinched, cold perspiration, and extremities numb. After the relief of the obstruction, the bowel became relaxed, and there was involuntary evacuations throughout entire illness.

One evening, tympanitis being marked and great distress arising from inability to start the gas, there being enormous distention in the region of duodenum, an enema of laudanum and starch was given. Within an hour the tension seemed to relax, there was a succession of loud reports and a flood of water; water, feces and gas came away simultaneously. Relief was immediate.

For two days the stools were swelled with pus. He was ill three weeks and was well advanced toward recovery before he regained control of the bowel. He is now perfectly well.

The other four cases present the same histories in general—one of the four being recurrent in form.

Gentlemen, I have wearied you. You may say that some of my cases might have recovered quicker under an operation. So they might. They might have died.

Again, I say, I do not decry operative measures. I only say enemata and, where indicated, laxatives are too valuable to be thrown aside, and that in the class of cases met with in general practice they are usually successful.

---

Papoid in Diphtheria.—

℞ Papoid ..................... gr. x.
Aquæ ..................... ℥ ss.
M. f. solution.

Kohts and Asch painted diphtheritic membranes with this solution every fifteen or twenty minutes with a soft brush. They found that the oftener the application was made the more rapidly membranes disappeared. Kohts treated several hundred cases by this method with the greatest success.

